# Chloride–hydrogen antiporters ClC-3 and ClC-5 drive osteoblast mineralization and regulate fine-structure bone patterning in vitro

**DOI:** 10.14814/phy2.12607

**Published:** 2015-11-24

**Authors:** Quitterie C Larrouture, Deborah J Nelson, Lisa J Robinson, Li Liu, Irina Tourkova, Paul H Schlesinger, Harry C Blair

**Affiliations:** 1 Department of Pathology, University of PittsburghPittsburgh, Pennsylvania; 2 Department of Neurobiology, Pharmacology & Physiology, University of ChicagoChicago, Illinois; 3 Departments of Pathology and of Microbiology, Immunology & Cell Biology, West Virginia University School of MedicineMorgantown, West Virginia; 4 Department of Cell Biology, Washington UniversitySaint Louis, Missouri; 5 Veteran’s Affairs Medical CenterPittsburgh, Pennsylvania

**Keywords:** Chloride–proton antiporter, ClC-3, ClC-5, mineral transport, osteoblast

## Abstract

Osteoblasts form an epithelium-like layer with tight junctions separating bone matrix from extracellular fluid. During mineral deposition, calcium and phosphate precipitation in hydroxyapatite liberates 0.8 mole of H^+^ per mole Ca^+2^. Thus, acid export is needed for mineral formation. We examined ion transport supporting osteoblast vectorial mineral deposition. Previously we established that Na/H exchangers 1 and 6 are highly expressed at secretory osteoblast basolateral surfaces and neutralize massive acid loads. The Na/H exchanger regulatory factor-1 (NHERF1), a pdz-organizing protein, occurs at mineralizing osteoblast basolateral surfaces. We hypothesized that high-capacity proton transport from matrix into osteoblast cytosol must exist to support acid transcytosis for mineral deposition. Gene screening in mineralizing osteoblasts showed dramatic expression of chloride–proton antiporters ClC-3 and ClC-5. Antibody localization showed that ClC-3 and ClC-5 occur at the apical secretory surface facing the bone matrix and in membranes of buried osteocytes. Surprisingly, the *Clcn3*^−/−^ mouse has only mildly disordered mineralization. However, *Clcn3*^−/−^ osteoblasts have large compensatory increases in ClC-5 expression. *Clcn3*^−/−^ osteoblasts mineralize in vitro in a striking and novel trabecular pattern; wild-type osteoblasts form bone nodules. In mesenchymal stem cells from *Clcn3*^−/−^ mice, lentiviral ClC-5 shRNA created *Clcn3*^−/−^, ClC-5 knockdown cells, validated by western blot and PCR. Osteoblasts from these cells produced no mineral under conditions where wild-type or *Clcn3*^−/−^ cells mineralize well. We conclude that regulated acid export, mediated by chloride–proton exchange, is essential to drive normal bone mineralization, and that CLC transporters also regulate fine patterning of bone.

## Introduction

In mammals, bone formation occurs behind a tight epithelium-like layer of osteoblasts that produce bone matrix components and regulate the environment for bone matrix assembly. The components are mainly type I collagen, calcium, and phosphate. These are produced by osteoblasts via well-studied mechanisms (Blair et al. 2007, 2011). However, sealed inside the physiologic bone biogenerator, the osteon, additional transport across the epithelium-like osteoblasts is critical for mineral deposition. Specifically, precipitation of hydroxyapatite from calcium and phosphate solutions in a sealed compartment liberates a massive amount of acid, equation 1.



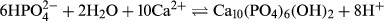
1

Equation 1 implies that, during mineral synthesis, by removing acid mineral precipitation in the matrix compartment can be driven effectively to completion regardless of extracellular conditions, since matrix is isolated from extracellular fluid. This holds as long as transport of soluble components into the matrix space, and of acid out, is maintained. Furthermore, the chemistry shown in equation 1 demands that bone *cannot* mineralize if the protons are not removed; mineral formation stops at pH below 5.6 (Neuman and Neuman 1958). Until recently, the mechanism of proton transport was entirely unknown, primarily because it was not studied systematically. Similarly, the mechanism of the reverse process, acidification to solubilize bone mineral by the osteoclast, was unclear until studied (Blair et al. 1989, 1991; Teti et al. 1989; Schlesinger et al. 1994).

Our recent studies of the mineralizing unit, or osteon, showed that sodium–hydrogen exchangers 1 and 6 (NHE1 and NHE6) are highly expressed at the basolateral surface of mineralizing osteoblasts (Liu et al. 2011), along with the PDZ-organizing protein sodium–hydrogen exchanger regulatory factor 1 (NHERF1). This makes the surface of the osteon a powerful organelle for secreting acid into the extracellular fluid (Liu et al. 2012). Complementing acid removal, NHERF1 enhances the local activity of the neutral phosphate transporter-2 (Npt2) providing the essential component phosphate to the osteoblast (Wang et al. 2013) for phosphate secretion, mainly as ATP and ADP; calcium moves by facilitated diffusion (Blair et al. 2011). To support bone mineralization in the osteon, a complete, high-capacity pathway for proton extraction from the matrix through the osteoblast is required; NHEs neutralize large cellular acid loads, but do not mediate H^+^ entry.

We studied expression of proton transporters in mesenchymal stem cells and in mineralizing osteoblasts by cRNA gene screens. It is established that ClC-5 (Picollo and Pusch 2005; Scheel et al. 2005) and ClC-3 (Guzman et al. 2013) function as chloride–proton exchangers, and the chloride–proton antiporter ClC-3 emerged as a key candidate supporting H^+^ entry into osteoblasts, along with large but lesser quantities of ClC-5. A role for CLCs, including ClC-3, in acidification of epithelial cells is established in other contexts (Claud et al. 2008). We studied the role of these transporters in bone mineralization using murine and human osteoblasts in vitro, and using *Clcn3*^−/−^ mice.

## Materials and Methods

### Genome-wide expression screening

Genome-wide expression screening was as described (Robinson et al. 2010), using isolated RNA to make double-stranded cDNA, from which biotin-labeled cRNA was made and hybridized to the DNA array on glass. The Hu145 133.2 probe set of 54,676 cDNAs, 20 replicates per target, was used (Affymetrix, Santa Clara, CA). Presence of transcripts and differences between treatments were determined from the signal and variation in each assay replicate, with statistical confidence indicated.

### ClC-3 knockout mice

Mice with a knockout allele replacing 13 bp of exon 6 and all of exon 7 with an insert including neomycin resistance were the kind gift of Fred Lamb, Vanderbilt University (Dickerson et al. 2002). The animals have a mixed 129/Sv and C57B1/6J background. Knockout, wild-type, and heterozygote littermates were used. For analysis of bone growth in situ, animals were labeled with 25 *μ*g/g body weight with calcein at 4 and 1 day prior to sacrifice, to show bone formation as fluorescent bands in frozen sections of bone as described (Liu et al. 2012).

### Human mesenchymal stem cells and osteoblasts

Cells, pretested media, and supplements (Bullet kit) were from Lonza (Walkersville, MD). The hMSCs used were from a 22-year-old female; human osteoblasts were from an 11-year-old female. All cell culture was at 37°C, in 5% CO_2_ humidified air, and media were replaced each 2–3 days unless noted. Cells were grown in Dulbecco’s modified Eagle’s medium (DMEM) with 5.5 mmol/L of glucose, l-glutamine, sodium pyruvate, 10% fetal bovine serum, 30 *μ*g/mL of ascorbic acid, 30 *μ*g/mL gentamicin, and 15 ng/mL amphotericin-B.

### Mouse MSC

Mouse MSCs were isolated as described (Liu et al. 2012). Bone marrow from 6-month-old mice was collected by flushing femora and tibiae with RPMI 1640, 12% FBS, and antibiotics. Disaggregated cells were filtered to remove clumps, and plated. At 3 h, nonadherent cells were washed off and replated. Cells then adherent on day 2 were washed and grown in mouse MesenCult medium with serum (StemCell Technologies, Vancouver, Canada). At 80%, confluence cultures were trypsinized and replated at 5 × 10^5^/cm^2^ and grown in DMEM with 5.5 mmol/L of glucose, FBS, and antibiotics. MSCs were used at passages 5–10. Osteoblast differentiation was induced by 30 *μ*g/mL ascorbic acid, 200 nmol/L hydrocortisone, and 10 mmol/L glycerol-2-phosphate, with FBS reduced to 10%.

### Lentivirus

A pLKO shRNA lentiviral plasmid for mouse was purchased as *Escherichia coli* bacterial stock (ClC-N5 TRC shRNA 69494, Sigma, Saint Louis, MO), containing the shRNA CCGGCCTATGATGATTTCAACACAACTCGAGTTGTGTTGAAATCATCATAGGTTTTTG, GFP, puromycin, and ampicillin resistance. A colony was isolated and grown in Luria broth with ampicillin; plasmid was isolated by alkaline lysis; endotoxin was removed by nonionic detergent phase separation (MiraCLEAN, Mirus-Bio, Madison, WI). Vector preparation and titration were as described (Sena-Esteves et al. 2004; Geraerts et al. 2005; Ravi et al. 2015), and packaged using commercial envelope and packaging plasmid preparations (Addgene, Cambridge, MA). High-efficiency plasmid delivery was obtained using TransIT-LT1 (Mirus-Bio, Madison, WI) in serum-free MEM (OptiMEM, Sigma) with 3 *μ*L of TransIT-LT1 per *μ*g of DNA, incubated with the plasmids for 30 min. This mixture was incubated with packaging cells, HEK293T at passages 2–5, in DMEM with 25 mmol/L of glucose and antibiotics, overnight. Medium containing viral particles was collected every 24 h and stored at 4°C, filtered through 0.45 *μ*m cellulose and concentrated with 15 kD retention centrifugal filters (Amicon, Millipore, Billerica, MA). Virus titer was determined by anti-p24 ELISA (Lenti-X p24 Rapid Titer, Clontech, Mountain View, CA).

### Viral transduction

Infection was as described (Wein et al. 2008). MSCs were transduced at 60–70% confluence with lentivirus in minimal medium volume 6 *μ*g/mL of 1,5-dimethyl-1,5-diazaundecamethylene polymethobromide (Polybrene, Sigma). Calculated multiplicity of infection was 9. Cells were placed in growth medium 24 h after the transduction. Cells were selected using 2.5 *μ*g/mL puromycin for 7 days, beginning 48 h after transduction.

### Protein extraction, western blots, and in situ labeling

Cells (2 × 10^6^) were lysed on ice for 5 min with RIPA buffer (10 mmol/L Tris, 1 mmol/L EDTA, 0.5 mmol/L EGTA, 1% Triton X-100, 0.1% sodium deoxycholate, 0.1% SDS, 140 mmol/L NaCl at pH 8), with proteinase and phosphatase inhibitors. Lysates were sonicated and cleared by centrifugation. Protein concentration was determined by bicinchoninic acid (Thermo Fisher) binding. After heating 5 min at 95°C in sample buffer, aliquots, 40 *μ*g or as stated, were separated on 12% SDS-polyacrylamide gels in Laemmli buffers. Proteins were transferred to polyvinylidine-derivitized nylon; unreacted groups were neutralized in 50 mmol/L Tris, 140 mmol/L NaCl, 0.05% polyoxyethylene-20-sorbitan laurate (Tween 20), pH 7.4 (TBST) with 5% nonfat dry milk overnight at 4°C. Membranes were rinsed with TBST and incubated with primary antibodies: goat polyclonal anti-ClC-5 D-17, recognizing a ClC-5-specific internal epitope (Santa Cruz, Santa Cruz, CA), 1:150, or rabbit polyclonal anti-ClC-3 raised to amino acids 80–125 of human ClC-3 (Bioss, Woburn, MA), 1:400, or mouse anti-*β*-actin (Sigma) 1:40,000 overnight at 4°C. Unbound antibody was washed off with TBST; secondary antibodies were added 1:40,000 for 1 h: horseradish peroxidase-conjugated (HRP) goat anti-rabbit IgG or HRP donkey anti-goat IgG (Jackson ImmunoResearch, Westgrove, PA), or HRP anti-mouse IgG (Sigma). The membranes were washed with TBST and protein bands were revealed by enhance chemoluminescence substrate (Life Technologies, Carlsbad, CA, USA). For in situ labeling of ClC-3/5, protocols used frozen sections of mouse bone and the anti-CLC primary antibodies as in western blots, and fluorescent second antibodies and photographed in an inverted fluorescence microscope using a 40× oil objective as described (Palagano et al. 2015). Sections of the mouse bone were fixed with cold acetone, decalcified with 10% sodium citrate; in this case, anti-ClC-3 was used at 1:100 dilution and anti-ClC-5 at 1:25. Secondary antibodies were donkey anti-goat Cy3 and donkey anti-Rabbit Alexa 488, both at 1:500 (Jackson ImmunoResearch, West Grove, PA and Invitrogen, Carlsbad, CA, respectively). Nuclei were stained with Hoechst 33342 (Thermo Fisher, Pittsburgh PA).

### RNA and DNA extraction and PCR

Messenger RNA was isolated by oligo (dT) affinity (RNeasy; Qiagen, Valencia, CA). First-strand cDNA was synthesized with Moloney murine leukemia virus reverse transcriptase (Superscript III, Life Technologies), and random hexamer primers, 10 mM DTT, and recombinant RNase inhibitor (RNaseOUT, Life Technologies). Quantitative RT-PCR was performed using cDNA as the template in 25 *μ*L reaction mixtures with premixed SYBR green, dNTPs, buffer, Taq DNA polymerase (SYBR Green Master Mix; Stratagene/Agilent, Santa Clara, CA) with 1 *μ*L of first strand cDNA and 250 nmol/L primers (Table1). Expression relative to mouse *β*-actin or human glyceraldehyde-3-phosphate dehydrogenase cDNA was calculated as described (Robinson et al. 2009). Product sizes were verified by electrophoresis on 2% agarose. Unless noted, PCR used 94°C denaturation for 2 min, 57°C annealing for 30 sec, and 72°C elongation for 1 min, for 36 cycles, and duplicate assays each run in duplicate were performed. DNA was isolated by homogenization in denaturing buffer and binding on silica glass columns (EZNA DNA/RNA Isolation, Omega BioTek, Norcross, GA). PCRs contained 1 *μ*L of DNA and 0.5 unit of Taq polymerase (Platinium Taq, Invitrogen, Carlsbad, CA).

**Table 1:** Primer sequences and predicted PCR products

Gene	GenBank	Forward	Reverse	bp
Actin	NM_007393	5′GATATCGCTGCGCTGGTCGTC	5′ACGCAGCTCATTGTAGAAGGTGTG	275
*Alp*	NM_007431	5′ATCGGAACAACCTGACTGACCCTT	5′ACCCTCATGATGTCCGTGGTCAAT	131
*Clcn3*	AF029347	5′CCAAGACCCCGCTTCAATAA	5′CGAGTCCCGCAGATTAAAGA	122
*Clcn5*	NM_016691	5′GAGGAGCCAATCCCTGGTGTA	5′TTGGTAATCTCTCGGTGCCTA	101
*Col1*	NM_007742	5′TTCTCCTGGCAAAGACGGACTCAA	5′AGGAAGCTGAAGTCATAACCGCCA	159
*Ocn*	NM_007541	5′ACCATCTTTCTGCTCACTCTGCTG	5′TATTGCCCTCCTGCTTGGACATGA	117
*Runx2*	NM_001145920	5′-ATGATGACACTGCCACCTCTGAC	5′ACTGCCTGGGGTCTGAAAAAGG	105

### Histomorphometry, histochemistry, and in situ labeling

Alkaline phosphatase activity was determined using 7-bromo-3-hydroxy-2-naphthoic-*O*-anisidide (naphthol AS-BI phosphate) substrate, reacted with fast blue-to-precipitate blue insoluble product, at pH 9.5 (leukocytes alkaline phosphatase kit, Sigma). Von Kossa silver stain for mineral used cell cultures fixed in 3.7% formaldehyde for 2 min. Mineral was stained with 2% AgNO_3_ under UV light for 10 min. Animals were labeled with 25 *μ*g/g of animal weight of calcein 4 days and 1 day prior to sacrifice. Frozen sections of vertebrae, 4-*μ*m thick, were obtained using carbide blades and a tape transfer system (CryoJane; Instrumedics, St. Louis, MO). Histomorphometric analysis was as described (Robinson et al. 2012).

### Statistics

Unless stated, the data are mean ± SD. Individual comparisons used Student’s *t*-test, multiple comparisons used analysis of variance, and *P *≤* *0.05 is reported as significant.

## Results

### The chloride/hydrogen exchanger ClC-3 is strongly expressed in mineralizing osteoblasts; ClC-5 occurs at significant levels

Previously we identified NHE1 and NHE6 as massively expressed sodium–hydrogen exchangers in the basolateral surface of mineralizing osteoblasts (Liu et al. 2011). These cells form the epithelium-like boundary on the osteon (Graphical Abstract); only this surface is exposed for release and efflux of protons to the extracellular compartment (Liu et al. 2012). The other surface of the osteoblasts, the apical membrane, abuts the bone matrix compartment. We hypothesized that it must support equal and regulated H^+^ influx from matrix into the osteoblast.

**Figure 1:**
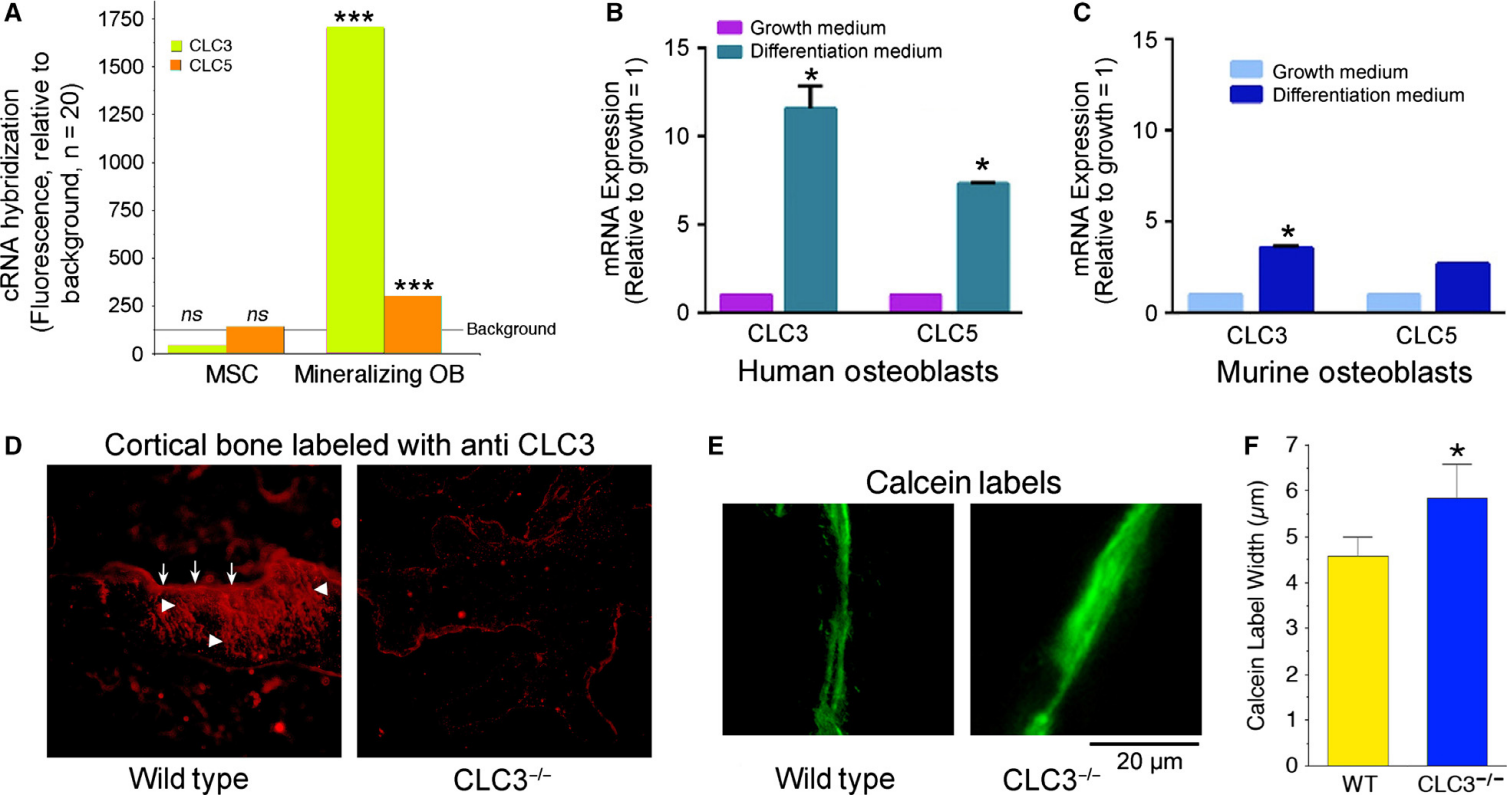
Expression of ClC-3 and ClC-5 in mineralizing osteoblasts, and the mild bone phenotype of the ClC-3 knockout mouse. (A) Microarray gene screens of mineralizing osteoblasts showed strong expression of ClC-3 and ClC-5, greatly increased over expression in precursor MSC. The black line labeled background is the median fluorescent signal of nonexpressed genes. This is extremely strong expression for this class of genes in many folds that of cells using ClC-3 in acid vesicles within the cells. Quantitative PCR confirmed amplification of ClC-3 and ClC-5 in mineralizing human osteoblasts relative to growing cells (B); ClC-3 and ClC-5 mRNAs increased with differentiation. ClC-3 and ClC-5 in murine MSC and osteoblasts (C) showed the same pattern of expression; in this case differences for ClC-5 did not reach significance (*P* = 0.09). In all cases the pattern of CLC expression confirmed gene screening (A). Larger changes in ClC-5 expression occurred in replicates and in *Clcn3*^−/−^ osteoblasts (see Fig.2F). (D) Fluorescent ClC-3 antibody labeling of mouse bone from wild-type and ClC-3 knockout animals. Each field is 350 *μ*m across, and shows a section of cortical bone. In the wild type (left) there is strong labeling in the surface layer of osteoblasts at the apical surface (arrows); labeling carries down into the canalicular system connecting the osteocytes and osteoblasts (arrowheads). That the unusual cell membrane pattern is actually ClC-3, and not artifact, shown by comparison with the ClC-3 knockout bone (right). The weak red at the bone periphery is an artifact due to the edge of the dense matrix. (E) Characterization of bone formation in the ClC-3 knockout mouse, in 3-month-old animals labeled with calcein 5 and 1 days before sacrifice, showed only minor effects, including slight broadening of the calcein labels. (F) Blinded measurements of interlabel distance showed a small, but significant, increase in the ClC-3 knockout animals. *N* = 20, mean ± SEM.

Using Affymetrix cRNA microarrays (Robinson et al. 2010) we compared expression for undifferentiated human MSC and osteoblasts (Fig.1A). There was, in the human cells, a striking change of ClC-3 expression in MSC, with a median signal in MSC of 29 with *P* value for expression from 0.12 to 0.35, to a median signal in mineralizing osteoblasts of 1800 with p values uniformly <0.0002. A second CLC, ClC-5, was also increased in mineralizing osteoblasts, but at lower levels, with convincing *P* values of 0.002–0.004. The same gene screens revealed the increases in NHE1 and NHE6 to about 1000 with *P* values of <0.0002 in osteoblasts, as reported (Liu et al. 2011, 2012). Other CLCs and other potential regulated inward proton transporters were not strongly expressed in human osteoblasts (not illustrated). We concluded that expression of ClC-3 and ClC-5, together with NHE1 and NHE6 at the basolateral membrane, might form the basis of a coordinated transcellular system to move H^+^ produced by precipitation of hydroxyapatite in the matrix. This approach assumes that the major mechanisms have a strong overlap in humans and mice used in subsequent work; this assumption was validated by PCR assays comparing the species (Fig.1B–C). In both human and murine osteoblasts, both ClC-3 and ClC-5 mRNAs, were increased in mineralizing cells, and subsequently were studied in murine MSCs and osteoblasts.

We followed this with in situ antibody labeling of ClC-3 in bone from *Clcn3*^+/+^ and *Clcn3*^−/−^ mice (Fig.1D). The ClC-3 labeling was consistent with osteoblast membrane expression of ClC-3 in surface osteoblasts and in the membranes of the canalicular system. Osteocytes buried in mineral, from earlier layers of bone formation, maintain processes in these canaliculi, connecting to the surface layer of osteoblasts synthesizing new matrix. In bone from *Clcn3*^−/−^ animals labeling was absent (Fig.1D, right), demonstrating that the strong ClC-3 labeling in the wild-type animal bone is not an artifact.

Surprisingly, the bone phenotype of *Clcn3*^−/−^ animals, at least at the 3-month’s age studied, was very mild. By visual inspection the bones of each animal appeared unremarkable and very similar; microcomputed tomography showed differences in the order of 5%, with a trend to lower bone volume, trabecular thickness, and increased trabecular spacing in the *Clcn3*^−/−^ relative to wild-type animals, but none of these reached significance (*n* = 8, not illustrated). These results were puzzling, since if ClC-3 is essential to acid transport an impressive phenotype would be expected. Bone formation in *Clcn3*^+/+^ and *Clcn3*^−/−^ animals was compared directly using calcein labeling of mineral deposition in vivo (Fig.1E–F). Blinded measurement of calcein double labels, done 5 and 1 days before sacrifice, showed that the *Clcn3*^−/−^ mice have anomalous bone mineral deposition, limited to blurring of mineral deposition. Note that broadened calcein lines reflect delayed mineralization rather than increased mineralization; nonmineralized matrix accumulates in bone mineralization defects including vitamin D deficiency. We hypothesized that the mild phenotype reflects that ClC-5 might compensate for ClC-3 loss.

### ClC-3 knockout osteoblasts have greatly increased ClC-5 expression

**Figure 2:**
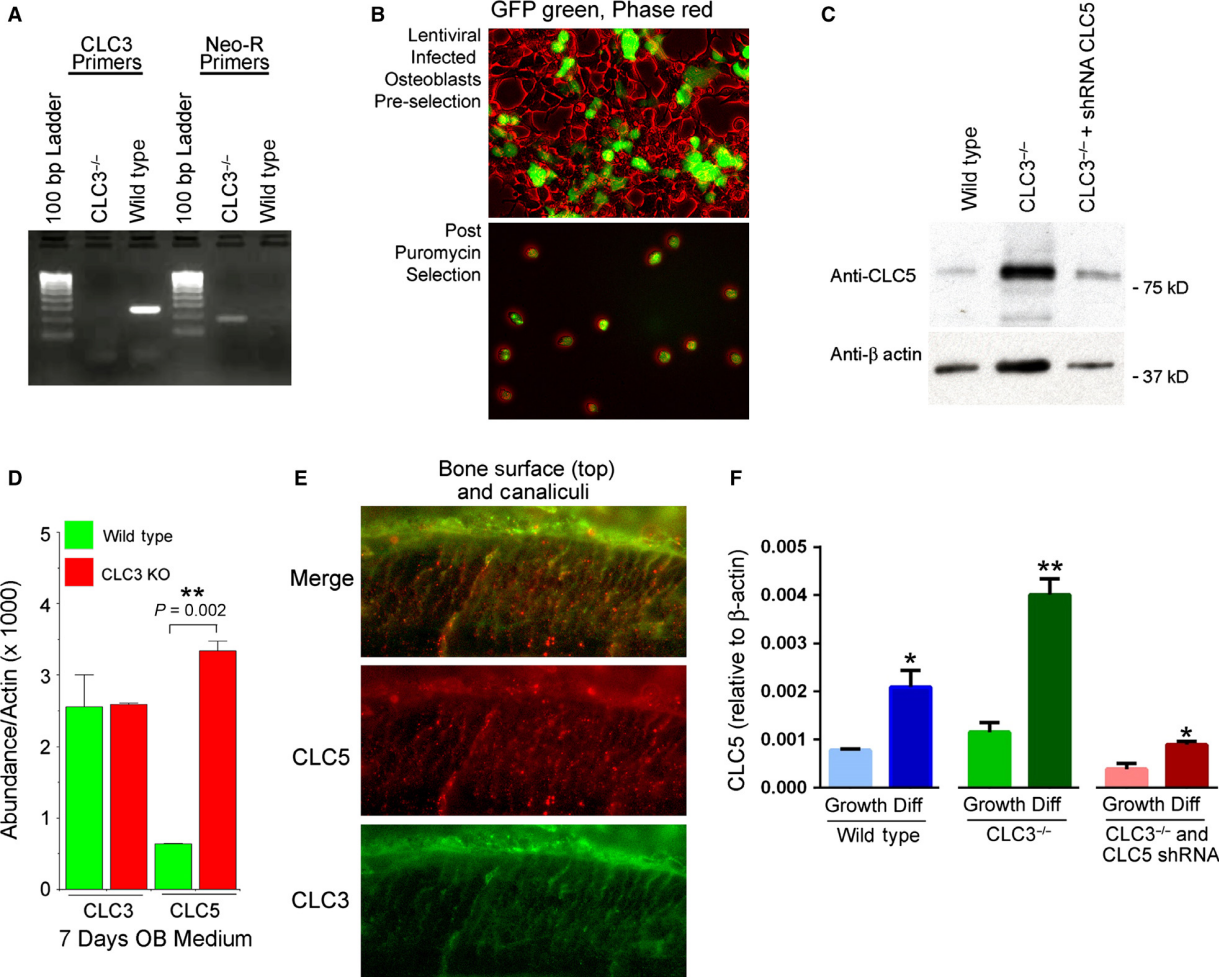
Preparation of ClC-3 and ClC-3/5 null mesenchymal stem cells. (A) PCR to probes in exons 6–7 showing complete removal in MSC from ClC-3 knockout animals. The neomycin control verifies presence of the insert (see text). (B) Green fluorescent protein to document lentiviral infection of MSC with plasmid carrying both GFP and ClC-5 shRNA. Initial infection was ∼25% efficient; puromycin selection increased the proportion of knockdown cells to quantitative (lower panel). However, shRNA degradation of target was ∼80% efficient, see PCR data following. (C) Western blot of ClC-5 wild-type and *Clcn3*^−/−^ MSC, with and without lentiviral knockdown of ClC-5, in osteoblast differentiation medium 2 weeks. The increase in ClC-5 in the ClC-3 null cells is not an artifact, see PCR assays following. With lentiviral shRNA, ClC-5 protein declined 80–90% relative to matched cultures without shRNA, to levels similar to wild-type ClC-5 expression (left). One of two western blots with similar results is shown. (D) Effect of ClC-3 KO on ClC-3 and ClC-5 mRNA expression. For ClC-3, primers for exons 1–2 detect a region unaffected by the knockout (in which a portion of exon 6 and exon 7 are deleted); this shows the amount of nonfunctional ClC-3 mRNA, which is not changed significantly in the knockout. The probe for ClC-5 amplifies a portion of exon 7; note the increase in ClC-3 knockout cells. The primers are listed in Table1. Mean ± range, *n* = 2 is shown, in one of two experiments with similar results. (E) Antibodies demonstrate that ClC-3 and ClC-5 occur in wild-type murine bone in essentially the same distribution, in the canalicular system, and at the basolateral surface of the osteoblasts. High power fields, 400 microns across. Distribution of ClC-3 is shown in green, ClC-5 in red, and the two merged are shown at the top. (F) Effect of lentiviral shRNA on ClC-5 in wild-type littermate and ClC-5 knockout MSC, in growth medium (left of each group) and after 1 week in differentiation medium. Differences relative to the growth medium controls, *P* < 0.05, *, of *P* < 0.01. One of two experiments is shown, each with *n* = 2.

Not having access to ClC-5 animals, we undertook study of bone formation by MSCs in culture. The MSCs from ClC-3 animals could then be treated with lentivirus shRNA to reduce ClC-5, to determine the effect of low expression of both CLCs on mineral deposition in culture. This might be necessary in any case, since if the function of CLCs is essential, the bone defect of ClC-3 and ClC-5 absence might be lethal. To confirm the genotype of MSCs from ClC-3 mice, the KO and wild-type (WT) alleles of ClC-3 were identified by PCR with genomic DNA using primers showing that the targeted region in exons 6–7 was absent in cell cultured from the knockout mice (Fig.2A), with targeting insert neomycin resistance shown as a positive control. Western blotting revealed low levels of ClC-3 protein in *Clcn3*^−/−^ mouse (not shown); defective protein is present, but nonfunctional (Dickerson et al. 2002). We purchased a plasmid with shRNA targeting ClC-5, containing a green fluorescent protein insert, puromycin and ampicillin resistance, and packaged it in lentiviral particles. Lentiviral infection was monitored by GFP fluorescence (Fig.2B). After initial infection, 25% of cells had GFP; after puromycin selection and 2 weeks in culture, all cells were GFP positive (Fig.2B, lower panel). In wild-type MSCs, ClC-5 was present at low levels (Fig.2C, left). However, in osteoblasts from *Clcn3*^−^/^−^ animals, ClC-5 protein was greatly increased by an unknown mechanism (Fig.2C, middle). After infection with the viral vector ClC-5 protein expression was suppressed by shRNA. This was not complete, but to levels similar to ClC-5 in wild-type cells (Fig.2C, right). These results were consistent with quantitative PCR in osteoblasts cultured for 1 week in osteoblast differentiation medium (Fig.2D). Wild-type controls infected with scrambled shRNA showed no difference relative to the uninfected wild type (not illustrated). That ClC-5 mRNA and protein is greatly amplified in *Clcn3*^−^/^−^ animals, which have a near normal bone phenotype (Fig.1E–F) suggested that ClC-5 might functionally compensate for ClC-3 loss. If this hypothesis is correct, ClC-5 and ClC-3 should occur in similar distribution in osteoblasts and osteocytes. This was the case, with antibody labeling of ClC-3 and ClC-5 in bone of normal animals having and overlapping distribution (Fig.2E). Thus, in the osteon, the two channels are codistributed and might compensate each other to a significant extent. Additional studies of ClC-5 mRNA in wild-type and *Clcn3*^−/−^ cells, in growth medium and in differentiation medium, with and without lentiviral ClC-5 shRNA confirmed greatly increased ClC-5 in *Clcn3*^−/−^ cells, and that the shRNA reduced ClC-5 by about 80% in either cell type (Fig.2F). C57 black mouse cells and wild-type *Clcn3*^+/+^ cells, mainly 129/Sv, showed the same pattern of ClC-3 and ClC-5 expression, with or without lentiviral ClC-5 knockdown, indicating that there is probably not a strong strain-specific effect on CLC expression in mice (not shown). The reason for the residual ClC-5 is uncertain, but likely is due to insufficient hsRNA to suppress the very strong ClC-5 expression. We next studied bone formation in *Clcn3*^−/−^ cells with and without ClC-5 hsRNA relative to bone formation in wild-type cells.

### Effect of eliminating ClC-3 and suppressing ClC-5 on bone formation in vitro

**Figure 3:**
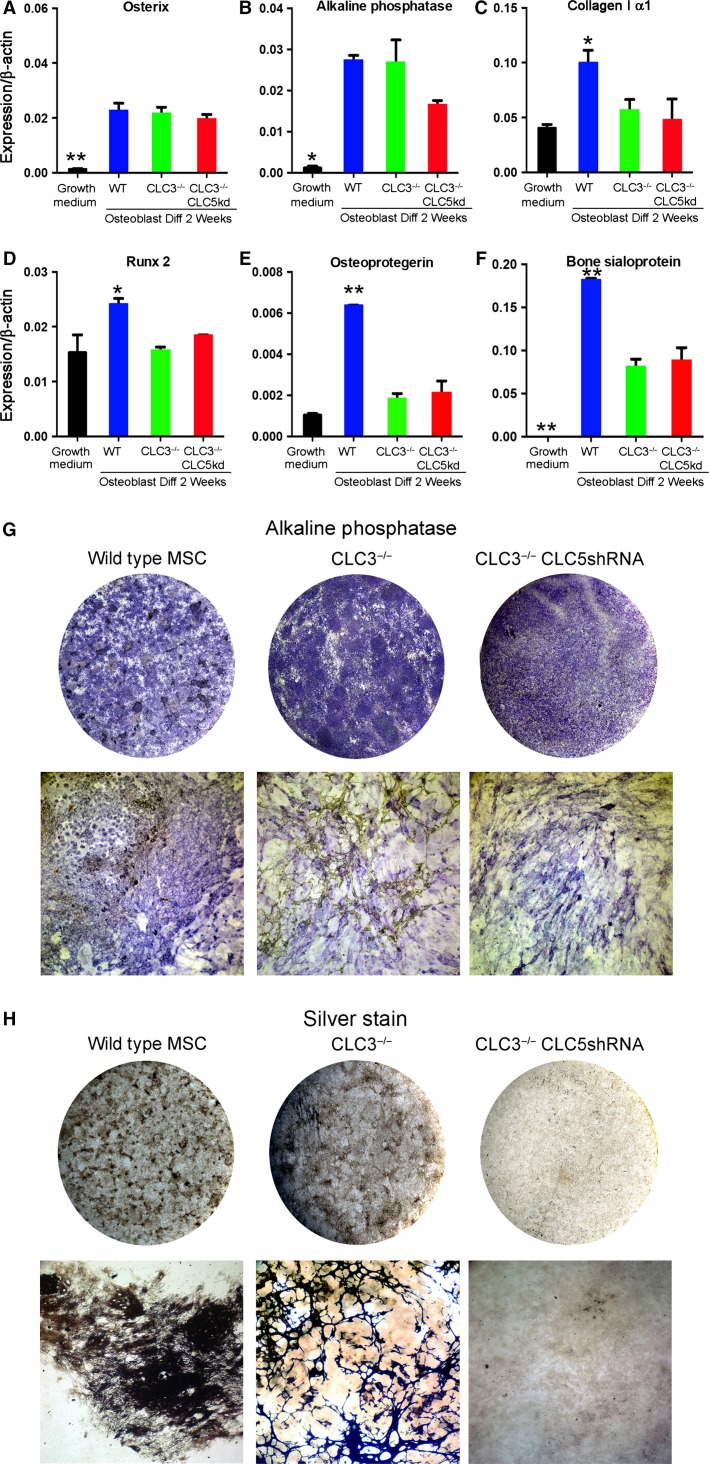
Effect of ClC-3 absence, and of ClC-5 knockdown in *Clcn3*^−/−^ cells, on bone matrix production and mineralization. (A–F) Quantitative PCR for major osteoblast proteins in growing wild-type cells and in wild-type, *Clcn3*^−/−^ cells, and *Clcn3*^−/−^ cells with lentiviral ClC-5 shRNA, at 2 weeks in osteoblast differentiation medium. (A) Osterix. (B) Alkaline phosphatase. (C) Col-Iα1. (D) Runx2. (E) Osteoprotegerin. (F) Bone sialoprotein. The proteins are expressed less in growing cells, and highest in wild type. In some, but not all, cases, expression is reduced in the knockout/knockdown cells, but remains higher than in the growing cells. (G–H) Alkaline phosphatase and mineral production in cell cultures. Round fields are whole wells of six-well plates; micrographs are 1.6 mm across. (G) Alkaline phosphatase activity was similar in wild-type cells or *Clcn3*^−/−^ cells with and without ClC-5 shRNA. Alkaline phosphatase distribution was more uniform in *Clcn3*^−/−^ ± ClC-5 knockdown cells. The lower panels are low power micrographs in cultures 14 days in osteoblast differentiation medium. (H) In whole cultures, mineral appeared nodular in the control, was more uniformly distributed in *Clcn3*^−/−^, cells, and was absent in the *Clcn3*^−/−^ cells with ClC-5 shRNA at 14 days in osteoblast differentiation medium. Low-power micrographs showed that the *Clcn3*^−/−^ cells had a remarkable trabecular pattern (see Fig.4). This pattern of bone differentiation was remarkably consistent in different isolates of *Clcn3*^−/−^ cells, in two MSC isolates and in several replicates of each isolate.

To understand the importance of increased ClC-5 expression in MSC cultured from ClC-3 mice we applied shRNA via lentivirus infection to reduce ClC-5 expression in bone-forming cultures from *Clcn3*^−/−^ mice, and characterized the resulting bone differentiation. The pretested shRNA-GFP-puromycin resistance construct was designed to target mouse ClC-5, and showed specific protein suppression during differentiation in our mouse osteoblast cultures (Fig.2F). Retention of 10–20% of the very high ClC-5 expression reflects a common effect with highly expressed genes, where a large amount of mRNA production and processing overwhelms the dicer system’s ability to degrade shRNA complexes, just as occurs in overwhelming viral infections. Notwithstanding that ClC-5 suppression was imperfect, the method made cells with suppressed ClC-5 similar to levels in wild-type cells, which we hypothesized would significantly affect bone differentiation in ClC-3 negative, ClC-5 knockdown cells on osteoblast differentiation. Analysis of bone-related mRNAs showed that expression is, as expected, increased by differentiation in osteoblast-promoting medium. Bone protein and promoter expression was highest in wild-type cells (Fig.3A–F). For the bone transcription factor osterix, expression was invariant with ClC-3/5 status; the transcription factor RunX2 was highest in wild-type cells. Alkaline phosphatase, osteoprotegerin, and bone sialoprotein were significantly increased over growing cells in wild-type and ClC-3/5 modified cells, but were mostly highest in the wild type. This may reflect in part different time courses of production in the different cell types: alkaline phosphatase, a durable ectoenzyme had strong and uniform activity at 2 weeks (Fig.3G) in wild-type and CLC-modified cells. Type I collagen was highly expressed including in all of the cell types; in any osteoblast culture a minority of cells are active in bone formation; relatively highly expressed proteins are less specific markers for that reason.

In contrast, by undertaking careful analysis of mineralization in culture, we uncovered a remarkable aspect of mineralization in *Clcn3*^−/−^ cells, possibly due to the increased ClC-5 expression in these cells (Fig.2). Specifically, mineral production was widespread and occurred in a distinct, fine trabecular pattern with sharp boundaries (Fig.3H, bottom middle panel). In spite of many attempts to formulate trabecular bone bioreactors, mineralization always has been in patchy, round, or spherical nodules and bone produced is not ideally suited for implantation. The highly branching trabeculae were a characteristic of the cells from ClC-3 mice and might indicate a practical method of creating trabecular bone in vitro (see Discussion); it occurred uniformly in ClC-3 cells from separate MSC isolations and in over a dozen separate tissue culture differentiation assays. In bone differentiation in vitro, formation of solid bone tissue has been inconsistent, unreliable (Jakob et al. 2012), and typically unimpressive even when best results are shown. Importantly, in keeping with the hypothesis that Cl/H exchange supports mineral deposition, mineral was uniformly absent in matched 2 week cultures of *Clcn3*^−/−^ cells with ClC-5 shRNA.

### Properties of bone matrix in CLC defective cells at high resolution

**Figure 4:**
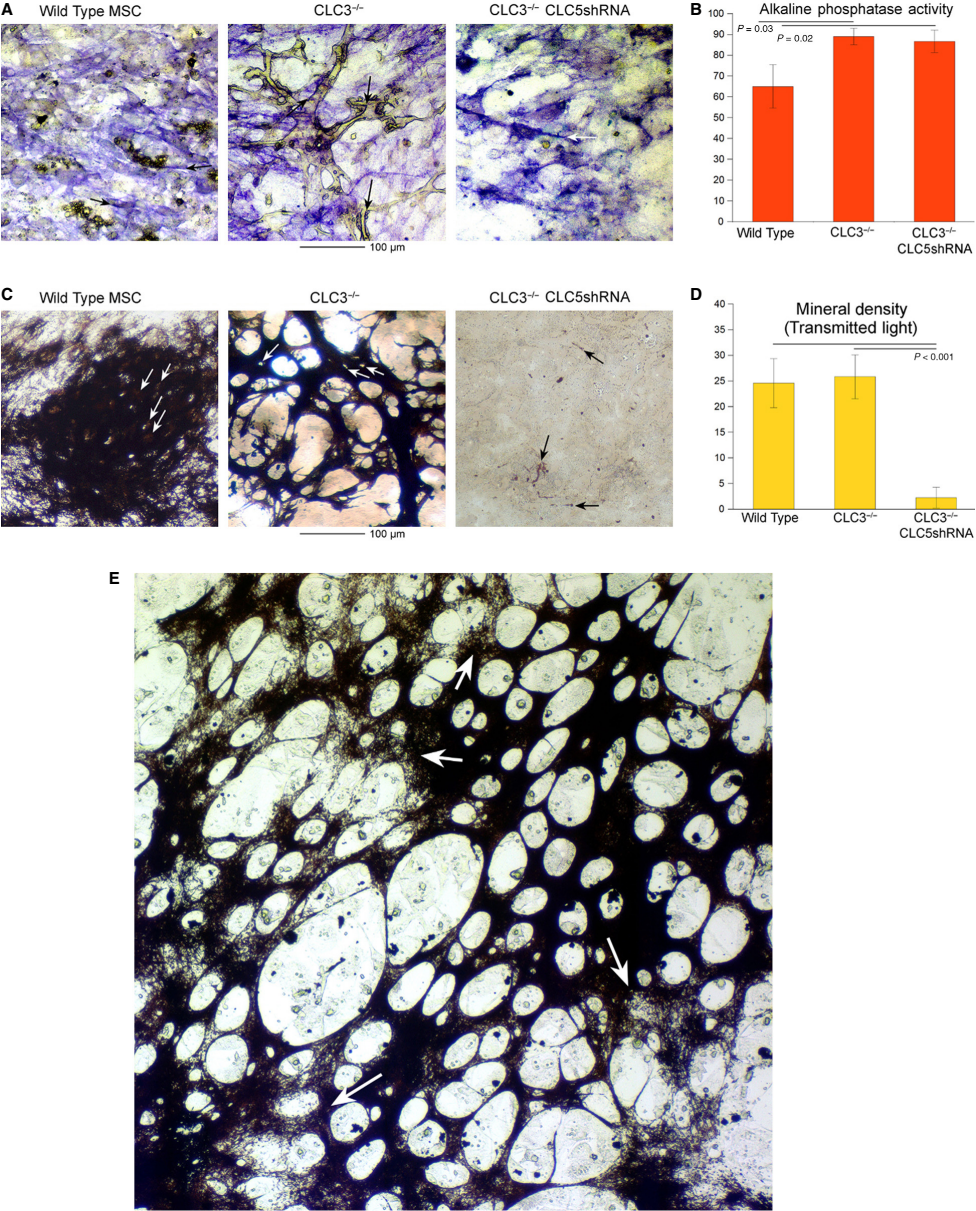
Characteristics of alkaline phosphatase and mineral deposition at high power in wild type, *Clcn3*^−/−^, and *Clcn3*^−/−^ with ClC-5 shRNA cells. All data for cultures are at 2 weeks in bone differentiation medium, except part G. (A) Alkaline phosphatase. Strong alkaline phosphatase activity occurs in fine linear areas consistent with cell surface and cell process-associated activity in wild-type (left), Clcn3^−/−^ (middle), and Clcn3^−/−^ and ClC-5 shRNA cells (right). In Clcn3^−/−^ cells there is dense alkaline phosphatase adjacent to much of the trabecular bone (refractile, unstained) (arrows, center panel). Fields are 250 *μ*m across. (B) Quantitative alkaline phosphatase activity at 3 weeks by densitometry of four replicates, similar to Figure3G–H upper panels. There was no difference between ClC-3 negative and ClC-3 negative ClC-5 shRNA cells. (C) Silver stain for bone mineral. In wild-type and in Clcn3^−/−^ cells, lacunae indicate that cells incorporated in the matrix. In the Clcn3^−/−^ and ClC-5 shRNA cells, no significant mineral occurs, although minor amounts of linear labeling are seen in the pattern similar to strong cell surface-associated alkaline phosphatase activity (A, right panel, arrows), suggesting nonspecific phosphate precipitation at sites of very high alkaline phosphatase (see text). Fields are 250 *μ*m across. (D) Densitometry for mineral, quantified as dark matter in transmitted light, in four replicates of the three cell types at 2 weeks in differentiation medium. There was no difference in overall absorbance in wild-type and *Clcn3*^−/−^ cells, despite the remarkable difference in matrix pattern. The *Clcn3*^−/−^ and ClC-5 shRNA cells made no meaningful mineral. (E) A cell culture of *Clcn3*^−/−^ cells as in (D) middle, but maintained in osteogenic medium for an additional week. The mineral area has expanded and now appears in sheet-like form with windows in irregular distribution. In this large area, it is clearly seen that some of the pattern is laid out but not fully mineralized. Magnification is the same as (D), with an expanded field, 750 *μ*m across.

Because of the remarkable trabecular nature of mineralization by the ClC-3 cultured MSCs we examined these cultures at high-magnification and quantified alkaline phosphatase activity and mineral density. In all cell types, alkaline phosphatase occurred in fine linear patterns consistent with cell surface-associated activity (Fig.4A), in keeping with its cell surface expression as established in other contexts (Magnusson et al. 1999). In the *Clcn3*^−/−^ cells dense alkaline phosphatase activity occurred adjacent the trabecular bone, in keeping with its distribution at the bone-attached osteoblast surface in vivo (Fig.4A, middle, arrows). Quantitative alkaline phosphatase activity showed minor increases in activity in the cells with modified CLCs, but no difference between *Clcn3*^−/−^ and *Clcn3*^−/−^, ClC-5 shRNA (Fig.4B).

Silver stain for bone mineral was strongly positive in wild-type and ClC-3 cells. At high power, gaps in the mineral were observed consistent with bodies of cells becoming osteocytes that are incorporated in the mineralized matrix (Fig.4C, left and middle panels). In *Clcn3*^−/−^, ClC-5 shRNA cells, no significant mineral as deposited. There were minor amounts of linear labeling at high power, in the morphology associated with the cell surface. We conclude that this most likely reflects that, in the presence of the glycerol-2-phosphate substrate at high concentration (10 mmol/L), some calcium–phosphate precipitate occurs outside of cellular mineral transport, similar to calcification of hypertrophic cartilage. Overall mineral deposition in wild-type and *Clcn3*^−/−^ cells was essentially the same at 2 weeks, notwithstanding the profound difference in the pattern of mineralized matrix. The *Clcn3*^−/−^, ClC-5 shRNA cells made no significant mineral. Following the mineralization in *Clcn3*^−/−^ cells for an additional week extended the mineral deposition to a sheet-like morphology with windows (Fig.4E), and it was clear that the fine patterning boundaries for bone formation were defined before mineralization was complete (arrows, Fig.4E).

## Discussion

A keyword search for idiopathic osteoporosis in the database Medline returns 800 reports that include a smattering of incomplete tubular acidosis and calcium wasting hypotheses, but in most cases have no rationale for the finding of osteoporosis. There is a clear problem with osteoporosis not fitting known categories of matrix defects. This likely reflects the existence of many mineral transport defects that, if not lethal, have mild or moderate phenotypes. Since humans are long-lived, defects accumulate with time to produce skeletal fragility, and cases may have multifactorial causes.

In an attempt to “close the loop” regarding one major incomplete system, acid elimination to drive mineralization to completion (Blair et al. 2011), we produced conclusive data that without ClC-3 and ClC-5, osteoblasts cannot mineralize in vitro. It is likely that for further in vivo studies, cell-specific knockouts may be required; mutations of *Clcn5* cause Dent’s disease (Silva et al. 2003) and constitutive *Clcn3/Clcn5* double knockout animals might be lethal if the expected severe skeletal mineralization defect occurs.

Precedents in bone include that ClC-7 mediates exchange of extracellular H^+^ for Cl^−^ and is required for normal osteoclast function (Kornak et al. 2001; Sobacchi et al. 2013). In addition, in a singular study (Wang et al. 2010), CLCs 3, 4, and 5 were hypothesized to drive osteoblast differentiation. Each CLC, when overexpressed in MC3T3-E1 osteoblast-like cells, localized to peripheral membranes and was associated with increased mineralization, in the mineralization pattern seen in normal wild-type cells. Furthermore, the same group recently showed that overexpressing ClC-3 in MC3T3-E1 rendered cells susceptible to mechanical force-induced upregulation of bone markers (Wang et al. 2015). Given the expression of ClC-3 in canaliculi of bone (Fig.1), and the nature of the proteins, this is not surprising and may be related to the role of ClC-3 in mediating formation of trabecular structures in differentiating bone (Figs.3, 4).

Much further work is required fully to characterize outward proton transport with bone mineral formation. The H^+^ gradient during mineral transport is minor, but “uphill”; bone matrix is maintained at about pH 7.6–7.8 (Blair et al. 2011). The extracellular chloride concentration is unknown, and precipitation of mineral may leave a hypotonic solution, requiring aquaporins or another water transport mechanism. Furthermore, a cation is required to drive Cl^−^/H^+^ exchange, since it is electrogenic. Potassium conductance is the likely candidate; a specific channel is not known, and there are many candidates. Potassium transport to drive H/Cl exchange, which is hypothetical, and some elements of osteoblast transport that are established, including the NHERF-1 regulatory protein, are omitted from the graphical abstract to make the main point clear. Additionally, chloride balance of the osteoblasts would be jeopardized by massive Cl/H exchange; a compensatory mechanism must exist, possibly a KCl symport. Proton balance is not a problem; transit of acid from the osteoblast is the function of NHE Na/H exchangers at the basolateral membranes of osteoblasts (Liu et al., 2011), as diagramed in the Graphical Abstract. Querying human mineralizing osteoblast cRNA screens showed high expression of both aquaporin and KCl symport transcripts (not illustrated); these will require specific study to validate the hypothesis that they fulfill support roles during bone synthesis in the isolated extracellular matrix compartment. There are many other specializations of osteoblasts, including high levels of expression of glutathione peroxidases and superoxide dismutases, which may be required to counteract free radical production during synthesis of bone matrix (not shown).

We began these studies in the belief that understanding the transport and regulatory mechanisms that support physiologic bone formation in the osteon will fundamentally change the prospect for patients who are categorized as having idiopathic osteoporosis. However, in the course of these studies we made an additional important observation: Osteoblasts, under conditions where the major Cl/H antiporter is knocked out and ClC-5 expression increased to compensate, make remarkably uniform trabecular bone in vitro (Fig.4C and E). A generation of attempts to make bone with suitable characteristics for surgical implants has fallen short. Despite numerous types of bioreactors containing many different types of supporting matrix or scaffold and mesenchymal stem cells, getting the artificial tissue to form meaningful three-dimensional bone has been a major challenge (Jakob et al. 2012). Problems include difficulties in MSC expansion and decoupling of cell growth and bone matrix formation. A cell line that produces trabecular bone reliably might greatly reduce the second problem, increasing efficiency and reducing dependency on scaffolds. Note, however, that *Clcn3*^−/−^ cells do not produce increased matrix relative to wild type, at least at the times studied. The difference is that bone production is widespread in a sheet of tissue with gaps, similar to trabecular bone in the spine. We do not yet have pathway data for why the major difference in trabecular pattern occurs. That, translating the result to human cells, and expansion of the cultures, remains to be done.
